# Cervical carcinoma following myelodysplastic syndrome: A case report

**DOI:** 10.3892/ol.2014.2061

**Published:** 2014-04-11

**Authors:** DU MENG, YAN-LAN CHAI, YIN-FANG HE, HONGLIAN HU, RUI LIU, ZI LIU

**Affiliations:** 1 Department of Radiotherapy Oncology, The First Affiliated Hospital of Xi’an Jiao Tong University, Xi’an, Shaanxi 710061, P.R. China; 2 Department of Gynaecology, Shangluo Central Hospital, Shangluo, Shaanxi 726000, P.R. China; 3 Department of Blood Pathology, The First Affiliated Hospital of Xi’an Jiao Tong University, Xi’an, Shaanxi 710061, P.R. China

**Keywords:** cervical carcinoma, myelodysplastic syndrome, treatment

## Abstract

Solid tumors following myelodysplastic syndrome (MDS) are rare and have no uniform treatment guidelines. The current study presents a rare case of a 47-year-old female diagnosed with cervical cancer (International Federation of Gynecology and Obstetrics stage IIIB) with an eight-year history of MDS. A multidisciplinary treatment discussion was organized and a rigorous treatment plan was developed. With injection of granulocyte colony-stimulating factor and interleukin-11 factor, transfusion of red blood cell suspension and close monitoring of the blood count, the patient was administered radiotherapy, specifically intensity modulated radiation therapy. However, a degree IV bone marrow suppression repeatedly assaulted, leading to interruption of the radiotherapy treatment. Eventually, the total dose received by point A (2 cm above the cervical os marker and 2 cm perpendicular to the uterine axis along the plane of the uterus) was 51 Gy. One month later, a gynecological examination and magnetic resonance imaging of the pelvis revealed that the treatment resulted in a complete remission. In conclusion, radiation therapy can still be implemented to obtain satisfactory local control when the hematopoietic function of the bone marrow is weakened due to long-term MDS.

## Introduction

Numerous cancer survivors are afflicted with long-term complications of cytotoxic treatment (1), of which the most critical one is the development of secondary neoplasia, including myelodysplastic syndrome (MDS). However, cases of solid tumors subsequent to or simultaneously with primary MDS have rarely been reported. A study by Kondo and Shinbo (2) reported a 62-year-old male suffering from MDS and gastric cancer. A study by Takahashi *et al* (3) reported a 66-year-old male suffering from MDS associated with synchronous double cancers of the stomach and the papilla of Vater. The current study presents the case of cervical cancer with an 8-year history of MDS. The study was approved by the ethics committee of The First Affiliated Hospital of Xi’an Jiao Tong University (Xi’an, China), and written informed consent was obtained from the husband of the patient.

## Case report

A 47-year-old female presented with a one-year history of substantial pale and malodorous vaginal discharge, occasional vaginal contact bleeding and contact pain. The patient denied all urinary and digestive symptoms. The patient had a previous MDS history for 8 years and was treated with multiple blood transfusions and oral retinoids, stanozolol and α-D3.

The Karnofsky performance status (4) score of the patient was 50. On physical examination, positive findings consisted of anemic appearance. A gynecological examination showed a cervix that was cauliflower-like with a diameter of 7 cm, covered with thick pus-moss. The tumor extended to the pelvic wall and the bilateral parametrial ligaments became thick and non-elastic. A IIIB stage was determined according to the International Federation of Gynecology and Obstetrics classification system (5).

The initial complete blood cell count was as follows: White blood cell (WBC) count, 0.9×10^9^ cells/l; red blood cell (RBC) count, 0.78×10^12^ cells/l; hemoglobin (HGB), 30 g/l; and platelet (PLT) count, 72×10^9^ cells/l; indicating the failure of bone marrow hematopoietic function. Laboratory studies revealed normal urine, feces, blood coagulation and liver and kidney function.

**Figure 1:**
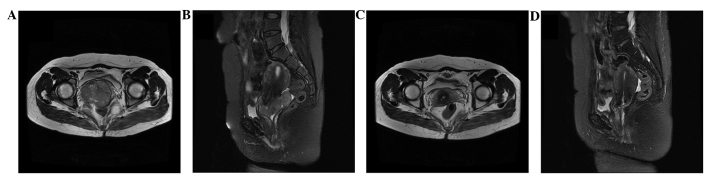
(A and B) Initial pelvic MRI showing a soft-tissue mass on the cervix, with a diameter of 7 cm, which invaded the bilateral parametrial ligaments and extended to the pelvic wall. (C and D) Pelvic MRI following treatment revealing that the cervical tumor had completely disappeared, indicating the efficacy achieved complete remission. MRI, magnetic resonance imaging.

**Figure 2:**
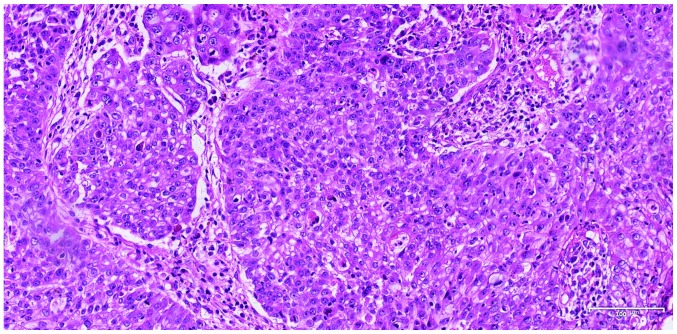
Histological analysis revealing a typical morphology of a low-grade (grade 3) squamous-cell carcinoma (hematoxylin and eosin stain).

**Figure 3:**
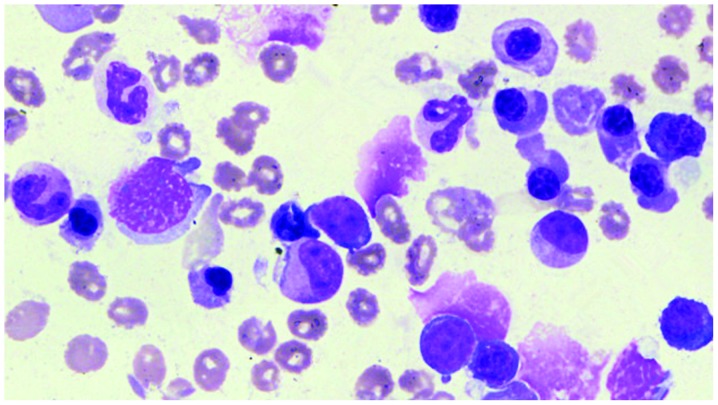
Bone marrow aspiration showing trilineage dysplasia and original cells increased (May-Giemsa stain, ×1,000).

No abnormality was found on the computed tomography scan of the chest and abdomen. Magnetic resonance imaging (MRI) of the pelvis (Fig. 1A and B) showed a soft-tissue mass on the cervix, which invaded the bilateral parametrial ligaments and extended to the pelvic wall. The imaging features supported the clinical diagnosis of cervical cancer stage IIIB. A colposcopy cervical biopsy was performed. The histological analysis revealed a low-grade (grade 3) cervical squamous-cell carcinoma (Fig. 2). A bone marrow aspiration in the ilium showed trilineage dysplasia and an increase in the original cells (Fig. 3).

A multidisciplinary treatment (MDT) discussion was conducted and a rigorous treatment plan was created. The peripheral blood count of the patient was improved. A total of 250 μg of granulocyte colony-stimulating factor (G-CSF) and 2 mg of interleukin-11 (IL-11) were subcutaneously injected twice a day to stimulate granulocyte and megakaryocyte proliferation and differentiation, respectively. RBC suspensions were transfused to correct the anemia. Hematopoietic materials, including vitamin B12 and folic acid, were also supplied to correct the anemia. External body radiation therapy (EBRT) was prescribed to the whole pelvic region in 25 fractions totaling 50 Gy, followed by intracavitary brachytherapy. The total dose of brachytherapy prescribed was 20 Gy/4 fractions [equivalent dose of 2 Gy/f (EQD2) = 25 Gy, α/β = 10] to point A (2 cm above the cervical os marker and 2 cm perpendicular to the uterine axis along the plane of the uterus). In order to protect the bone marrow, the EBRT was applied through intensity-modulated radiation therapy (IMRT) techniques and the volume of the pelvic bone receiving 20 Gy (V20) was controlled to <70% (6). Due to a recurrent III-IV degree myelosuppression, the chemotherapy was not included in the treatment regimen.

Radiation therapy was started when the WBC count, PLT count and HGB were ≥4×10^9^ cells/l, ≥50×10^9^ cells/l and ≥50 g/l, respectively. During the radiotherapy, the patient continued to have injections of G-CSF and IL-11 along with the intermittent transfusion of the RBC suspension. However, the patient experienced a IV degree myelosuppression following 16 Gy/8 fractions of EBRT. The EBRT was halted and brachytherapy, with a potential to produce weaker bone marrow suppression, was initiated. Following 20 Gy/4f of brachytherapy (EQD2 = 25 Gy, α/β = 10), the EBRT was continued. Subsequently, the amount of EBRT was pushed to 26 Gy/13 fractions. However, the WBC count, PLT count and HGB decreased to 0.35×10^9^ cells/l, 17×10^9^ cells/l and 29 g/l, respectively. The radiotherapy was withheld due to the recurrent IV degree myelosuppression and the failure of supportive care for the bone marrow hematopoietic function. Therefore, the total dose of point A was 51 Gy (EQD2, α/β = 10). The patient was then discharged from the hospital, as no further treatment was accepted. One month later, a gynecological examination and pelvic MRI (Fig. 1C and D) revealed the treatment effect was complete remission (CR) (7).

Seven months later, however, lung and liver metastasis occurred. The patient succumbed to an acute intracranial hemorrhage due to thrombocytopenia (PLT count, 9×10^9^ cells/l) 13 months after radiotherapy.

## Discussion

According to the National Comprehensive Cancer Network guidelines (8) for cervical cancer, the standard therapeutic schedule of a IIIB stage cervical cancer was concurrent chemoradiotherapy. However, the disease management should always be tailored according to the circumstances of the individual patient. For the present patient, a long-term bone marrow suppression caused by the MDS already existed, which excluded the standard chemoradiotherapy as a feasible choice. Following an MDT discussion, a plan of palliative radiotherapy was agreed upon. In order to protect the hematopoietic function of the bone marrow, the IMRT technique was applied to minimize the receiving dose of the pelvic bone marrow. A study by Rose *et al* (6) demonstrated that the hematological toxicity increased with an increasing pelvic bone marrow irradiation volume. Therefore, efforts should be made to maintain a V20 of >76%, which may reduce the hematological toxicity (6). For the present patient, the V20 was controlled to stay >70% to minimize the receiving dose of the pelvic bone marrow. Nevertheless, recurrent attacks of IV degree myelosuppression eventually led to the termination of radiotherapy. However, the treatment effect still achieved CR.

Using methods to control cancer (such as chemotherapy and radiotherapy) is likely to cause bone marrow damage, resulting in blood disease and a poor prognosis. However, the patient in the present case would have had a poor prognosis if the cancer was ignored. On the premise of elevating blood count and protecting the bone marrow, the patient in the present study accepted reduced-dose radiotherapy without chemotherapy. Finally, the total dose accepted by point A was 51 Gy. The treatment effect achieved CR. Therefore, for these patients to achieve the optimal therapeutic effect and the longest survival time, the dose of radiotherapy should be appropriately reduced on the premise of elevating blood count and protecting the bone marrow.

Anemia has a negative impact on local control, disease-free survival and overall survival rate of cervical cancer (9,10). In the present study, anemia caused by MDS persisted during the radiotherapy, which was a significant factor for progression-free survival (PFS). Insufficient radiation and chemotherapy, and low immunity caused by the long-term MDS were also involved in the poor prognosis of the cervical cancer. By contrast, the bone marrow cell loss induced by radiation was a poor prognostic factor for the MDS. Overall, the interaction between the two diseases eventually led to a shorter PFS and eventually a fatality due to intracranial bleeding.
